# Irisin Modulates IFNT Signaling in Bovine Luteal Cells

**DOI:** 10.1002/mrd.70043

**Published:** 2025-08-04

**Authors:** Ana Paula da Silva, Karine de Vargas Aires, Suzana Rossato Feltrin, Leonardo Guedes de Andrade, Julia Vieira Cambuí, Carlos Miguel Staudt, Luis Fernando Schütz, Christopher Price, Valério Marques Portela, Alfredo Quites Antoniazzi

**Affiliations:** ^1^ Graduate Program of Veterinary Medicine Federal University of Santa Maria Santa Maria Rio Grande do Sul Brazil; ^2^ Departament of Agriculture, Veterinary, and Rangeland Sciences University of Nevada Reno Nevada USA; ^3^ Centre de recherche en reproduction et fertilité, Faculté de médecine vétérinaire Université de Montréal St‐Hyacinthe Quebec Canada

**Keywords:** adipokines, corpus luteum, IFNT signaling, irisin

## Abstract

Adipokines, bioactive proteins derived from adipose tissue, serve as intermediaries between energy status and reproduction, playing pivotal roles in ovarian physiology. Among them, members of the Fibronectin Type III domain‐containing the (FNDC) family, including FNDC4 and FNDC5 (the precursor of irisin), have emerged as key modulators of fertility. This study investigated the expression of *FNDC4*, *FNDC5*, and their receptors (*ITGB1*, *ITGAV*, *ADGRF5*) across different stages of bovine CL lifespan and evaluated irisin's effects on interferon tau (IFNT)‐mediated signaling in bovine luteal cells. The relative abundance of *FNDC4* and *FNDC5* mRNA was significantly greater in Early II, Middle, and Late phases when compared to the Early I. The expression of *ITGB1* and *ADGRF5* in bovine CL was significantly greater in the Early II, Middle, and Late phases than in the Early I phase, and the abundance of *ITGAV* mRNA did not differ between the evaluated phases. Also, it was observed that mRNA expression of all evaluated receptors was greater in CL of nonpregnant cows on Day 18 compared to pregnant cows. In vitro experiments demonstrated that irisin treatment enhanced interferon‐stimulated genes (ISGs) such as *MX1* and *MX2* in luteal cells, suggesting irisin potentiates IFNT signaling. Notably, irisin did not alter steroidogenic enzyme expression or affect cell viability, proliferation, or apoptosis markers, indicating its effects are specific to IFNT signaling pathways. The present study shows that the genes *FNDC5* and *FNDC4* are expressed in the bovine corpus luteum at different stages of development and that irisin increased ISGs expression in bovine luteal cells in vitro. The effects of irisin on CL function may be indirect, as plasma irisin increases during the postpartum negative energy balance in dairy cows. We propose that this is part of a compensatory mechanism to modulate IFNT signaling in CL cells and indirectly improve embryonic signaling mechanisms.

## 1 Introduction

The corpus luteum (CL) is a temporary endocrine structure that is essential for maintaining pregnancy in mammals. Energy status is known to affect reproduction in mammals (Dupont et al. 2018). However, how energy balance affects pregnancy maintenance remains to be fully elucidated. Adipokines are bioactive proteins produced by adipose tissue that represent a link between energy and reproduction: their circulation in the blood is affected by energy status (Mellouk et al. 2017; Reverchon et al. 2014; Thilakan and Venugopal Bhuvarahamurthy 2023). Recent research has highlighted their significant role in fertility modulation and the development of reproductive disorders (Estienne et al. 2019, 2020; Nikanfar et al. 2021). These proteins impact ovarian physiology and their presence, as well as the presence of their receptors, has been detected in various ovarian compartments, including granulosa, theca, and luteal cells (Kurowska et al. 2018). Several studies have reported effects of adipokines on function of ovarian follicular cells (Daudon et al. 2023, 2025; Kurowska et al. 2018), but effects of these proteins on physiology of luteal cells remain to be elucidated.

The functionality of the CL depends on a complex interplay of luteotrophic and luteolytic signals, among which interferon tau (IFNT) plays a fundamental role. During early gestation, IFNT, secreted by the conceptus, induces classic interferon‐stimulated genes (ISGs) such as interferon‐stimulated gene 15 (*ISG15*), MX dynamin‐like GTPase 1 (*MX1*), MX dynamin‐like GTPase 2 (*MX2*), and other genes in the endometrium (Forde et al. 2011; Hansen et al. 2017). Beyond its paracrine effects on the endometrium, IFNT also acts directly on the CL (Romero et al. 2015). IFNT has been shown to modulate luteal cell function by upregulating ISGs, potentially supporting luteal maintenance and survival during early pregnancy (Hughes et al. 2020; Basavaraja et al. 2021; Meidan and Basavaraja 2022). These direct actions include inhibition of luteolytic pathways and promotion of luteotrophic gene expression, highlighting the CL as a direct target of conceptus‐derived IFNT signaling (Antoniazzi et al. 2013; Oliveira et al. 2008). However, how adipokines interact with IFNT‐mediated signaling and whether this interaction impacts CL function is unknown.

Members of the Fibronectin Type III domain‐containing (FNDC) family have recently been implicated in reproductive function (Daudon, Bigot, et al. 2022). Irisin, a bioactive peptide derived from the cleavage of the transmembrane protein FNDC5, is secreted by skeletal muscle and adipose tissue and influences physiological processes by acting on integrin receptors like ITGAV and ITGB1, enhancing glucose uptake and insulin sensitivity (Chen et al. 2016; Kim et al. 2018). Similarly, FNDC4, another related protein, is cleaved into an active form that modulates insulin sensitivity and acts through the ADGRF5 receptor, found in ovarian tissue (Liu et al. 2022).

Observations show that members of the FDNC family are particularly important for providing a link between energy status and female fertility. For example, plasma concentrations of irisin increase during the post‐partum period in dairy cattle (Daudon, Ramé, et al. 2022), and addition of irisin to bovine granulosa and theca cells in vitro inhibits steroidogenesis and/or metabolism (Daudon et al. 2023, 2025). The effect of FNDC family proteins on luteal cells remains unknown. This study aimed to identify the presence of FNDC4 and FNDC5 and their receptors at different stages of bovine CL lifespan. Furthermore, we evaluated the effects of irisin on the luteal response to IFNT using a primary culture model of bovine luteal cells.

## 2 Materials and Methods

### 2.1 Hormones and Reagents

Recombinant human irisin (aa 32–143; AG‐40B‐0136; Adipogen Life Sciences, USA; 100% homology with the first 126 aa of the bovine sequence) was used in the experiments. Recombinant ovine interferon tau (roIFNT) was kindly provided by Dr. Fuller W. Bazer, Texas A&M University.

### 2.2 Female Reproductive Tract Examination and Corpora Lutea Collection

Female reproductive tracts were obtained in a local slaughterhouse. After macroscopic observation of ovarian follicles (size and shape), CL (size, color, cavities, and vacuoles), cervix, and uterus, the CL were classified as previously reported (Miyamoto et al. 2000) into the following stages of the estrous cycle: Early I (Days 3–4, *n* = 10), Early II (Days 5–7, *n* = 10), Middle (Days 8–12, *n* = 10) and Late (Days 13–18, *n* = 10). In addition, groups of pregnant and nonpregnant stages were formed: Group P (CL of pregnant cows on Day 18, *n* = 10), and Group NP (CL of nonpregnant cows on day 18, n = 10). For this group, the CL samples were collected according to a previous in vivo study from our group (Manta et al. 2024). In the present experiment, however, all cows were randomly assigned, and the groups consisted of inseminated pregnant cows and non‐inseminated, nonpregnant cows. Following collection, CL samples were first frozen in liquid nitrogen and then frozen at −80°C until further processing for RNA isolation.

### 2.3 Experimental Design

To investigate the physiological relevance of irisin action on IFNT signaling, we used an in vitro model of bovine luteal cells treated with recombinant human irisin associated with treatment with roIFNT as described below in Section 2.4. Initially, bovine luteal cells were distributed into six groups as follows: Control (0 ng/mL of Irisin + 0 ng/mL of roIFNT); roIFNT (0 ng/mL of Irisin + 1 ng/mL of roIFNT); roIFNT + irisin1 (1 ng/mL of Irisin + 1 ng/mL of roIFNT); roIFNT + Irisin10 (10 ng/mL of Irisin + 1 ng/mL of roIFNT); Irisin1 (1 ng/mL of Irisin + 0 ng/mL of roIFNT); and Irisin10 (10 ng/mL of Irisin + 0 ng/mL of roIFNT). After 12 h of cell culture, luteal cells were treated with irisin for 12 h before IFNT treatment for 6 h. Then, cells were harvested and stored at −80°C for subsequent RNA extraction. The experiment was replicated four times, using six CL per replicate. The chronological treatment protocol (12 h with irisin followed by 6 h with roIFNT) was selected to better reflect the physiological sequence of events during early pregnancy, in which systemic exposure to irisin precedes conceptus‐derived IFNT signaling. All experimental groups, including those not treated with roIFNT, were maintained in culture for the total period to ensure consistency and comparability among treatments.

### 2.4 Primary Culture of Bovine Luteal Cells

Bovine ovaries were collected from a local slaughterhouse and transported to the laboratory in 0.9% saline containing penicillin (100 IU/mL) and streptomycin sulfate (50 µL/mL) at 4°C. Upon arrival, CL were washed with 0.9% saline and 70% alcohol both at 4°C, until the solutions became clear. CL were selected according to their morphological characteristics into early (1–6 days post ovulation), intermediate (8–12 days post ovulation), and late (15–17 days post ovulation) (Miyamoto et al. 2000). Early or intermediate CL selected were 15–25 mm in diameter, had luteal tissue present on the ovarian surface, were bloody to pink, light brown, or orange in color, and had a compact, soft consistency. Luteal cell culture was adapted from a previous protocol published by Pate (1993). Briefly, CL were mechanically separated from their fibrous capsule and washed in 70% alcohol to avoid contamination. Decapsulated luteal tissue was stored in PBS solution on ice until it was cut into smaller fragments with scalpel blades and then dissociated in 1% collagenase Type I (Sigma‐Aldrich, St Louis, MO, USA) and DMEM‐F12 solution 1% antibiotics (penicillin 10,000 IU/mL and streptomycin 10,000 μg/mL) for 45 min at 37°C, with vortexing for 1 min every 10 min. Then collagenase was inactivated with DMEM‐F12 solution supplemented with 10% FBS and 1% antibiotics (penicillin 10,000 IU/mL and streptomycin 10,000 μg/mL). The solution was filtered with 0.7 µm filters to obtain cells from the CL to reduce contamination of the culture. Then, the cells were resuspended in DMEM‐F12 solution supplemented with 1% antibiotics (penicillin 10,000 IU/mL and streptomycin 10,000 μg/mL) for 10 min. After discarding the supernatant, 2 mL of red blood cell buffer (Lysing Buffer R757 Sigma) was added and homogenized for 1 min. Then, the suspension was centrifuged 3× for 10 min at 200*g* in DMEM‐F12 solution containing 1% antibiotics. Cells were counted with 0.4% trypan blue solution (Sigma‐Aldrich, St Louis, MO, USA) at a dilution of 1:100. The experiment was represented by four independent replicates, using six different CL in each replicate, from ovaries collected on different occasions.

The CL cells exhibited 80% viability (trypan blue exclusion). After assessing their viability, the cells were plated out (200,000 cells/well) in 24‐well plates in 500 µL DMEM‐F12 supplemented with 10% FBS and 1% antibiotics and incubated at 37°C in 5% CO_2_ for 12 h. After these initial 12 h, the medium was discarded and replaced with 400 µL/well of DMEM‐F12 medium without FBS with 1% antibiotics (penicillin 10,000 IU/mL and streptomycin 10,000 μg/mL) and left in the incubator. Then, the cells were serum‐starved for 6 h to minimize metabolic interference, ensuring that the observed effects were due to the treatment with irisin and roIFNT. Irisin was added for 12 h, followed by roIFNT treatment. Cells were treated according to the groups described above and harvested 6 h after roIFNT addition and stored at −80°C.

### 2.5 RNA Extraction and RT‐qPCR

Total RNA was extracted using PureLink RNA Mini Kit (Thermo Fisher Scientific, Waltham, MA, USA) according to the manufacturer's instructions and quantified at a wavelength of 260 nm using a spectrophotometer (NanoDrop1000, Thermo Scientific, Wilmington, DE, USA). Total RNA (100 ng) was reverse‐transcribed (RT) using the iScript cDNA Synthesis Kit (Bio‐Rad, Des Plaines, IL, USA) at 25°C for 5 min and 46°C for 30 min. The reaction was terminated by incubation at 95°C for 5 min. Real‐time qPCR was performed using the CFX384 Real Time system (Bio‐Rad Laboratories, Hercules, CA, USA) using GoTaq DNA Polymerase (Promega, Madison, WI, USA) and specific primers (Table 1). After an initial denaturation step at 95°C for 3 min, 40 cycles were performed at 95°C for 10 s, followed by 1 min at 60°C to amplify each transcript. The reaction was carried out in duplicate. Data were normalized to a calibrator sample to quantify relative gene mRNA abundance using the ΔΔCt method with correction for amplification efficiency (Pfaffl 2001). Briefly, the target gene amplification Ct was normalized to the average mRNA abundance level of *RPL19* and *GAPDH* housekeeping genes, according to the ratio, *R* = housekeeping ECt/target ECt, where *E* is the amplification efficiency for each primer pair. It evaluated the expression of mRNA of steroidogenic enzymes (*P450sCC* and *3βHSD*), ISGs (*ISG15, MX1*, and *MX2*), and adipokines receptors genes (*ITGB1*, *ITGAV*) (Table 1).

**Table 1:** Primers for quantitative real‐time PCR analysis.

Target	Primer sequence	GenBank
*ISG15*	F: GGTATCCGAGCTGAAGCAGTT	NM_001009735.1
	R: ACCTCCCTGCTGTCAAGGT	
*MX1*	F: GTACGAGCCGAGTTCTCCAA	NM_173940.2
	R: ATGTCCACAGCAGGCTCTTC	
*MX2*	F: CTTCAGAGACGCCTCAGTCG	NM_173941.2
	R: TGAAGCAGCCAGGAATAGT	
*IFNAR1*	F: GAATCAGCTCTACCCGCTAAT	NM_174552.2
	R: GCTCTGGCTTTGACACAATAC	
*IFNAR2*	F: AGCCAGAATGTGTCAGCGAT	NM_174553.2
	R: AGAACAGGCGCAACATACGA	
*3βHSD*	F: GCCCAACTCCTACAGGGAGAT	NC_037330.1
	R: TTCAGAGCCCACCCATTAGCT	
*P450sCC*	F: CTTGCACCTTTCTGGCTAGG	NC_037348.1
	R: AAGGGGAAGAGGTAGGGTGA	
*FNDC5*	F: GGTAAGCTGGGATGTCTTGG R: CTGACCCTGGATGGATATGG	NM_001105421.1
*FNDC4*	F: GGCAACGTGTGATCCGAGAG R: AGAGTTCGGAAGTGCACCCT	NM_001102324.1
*ITGB1*	F: TGAGGTGAACAGCGAAGACA R: TTGCACTCACACACTCGACA	NM_174368.3
*ITGAV*	F: TTCGGCTATTCCATGAAAGG R: AGGCAGAGGGCAGGTTTTAT	NM_174367.1
*ADGRF5*	F: TATCCTGAGAATGTCGGTCAGACT R: TCGTACGTCACAACCACACT	NM_001193243.1
*GAPDH*	F: GCCATCAATGACCCCTTCAT R: TGCCGTGGGTGGAATCA	NM_001034034.2
*RPL19*	F: CCGGCTGCTTAGACGATACC R: CCGCTTGTTTTTGAACACGTT	NM_001040516.1

Abbreviations: F, forward primer; R, reverse primer.

### 2.6 Statistical Analysis

The data that did not follow a normal distribution (Shapiro–Wilk test) were transformed to logarithms. Homogeneity of variance was tested with O'Brien and Brown‐Forsythe tests. Analysis was performed with JMP software (SAS Institute) with treatment as main effect and culture replicate as a random variable in the *F*‐test. Differences between means were tested with the Tukey–Kramer HSD test. Data are presented as means ± SEM. Statistical significance was considered at *p* < 0.05.

## 3 Results

### 3.1 FNDC4 and FNDC5 mRNA Expression in Bovine CL

**Figure 1:**
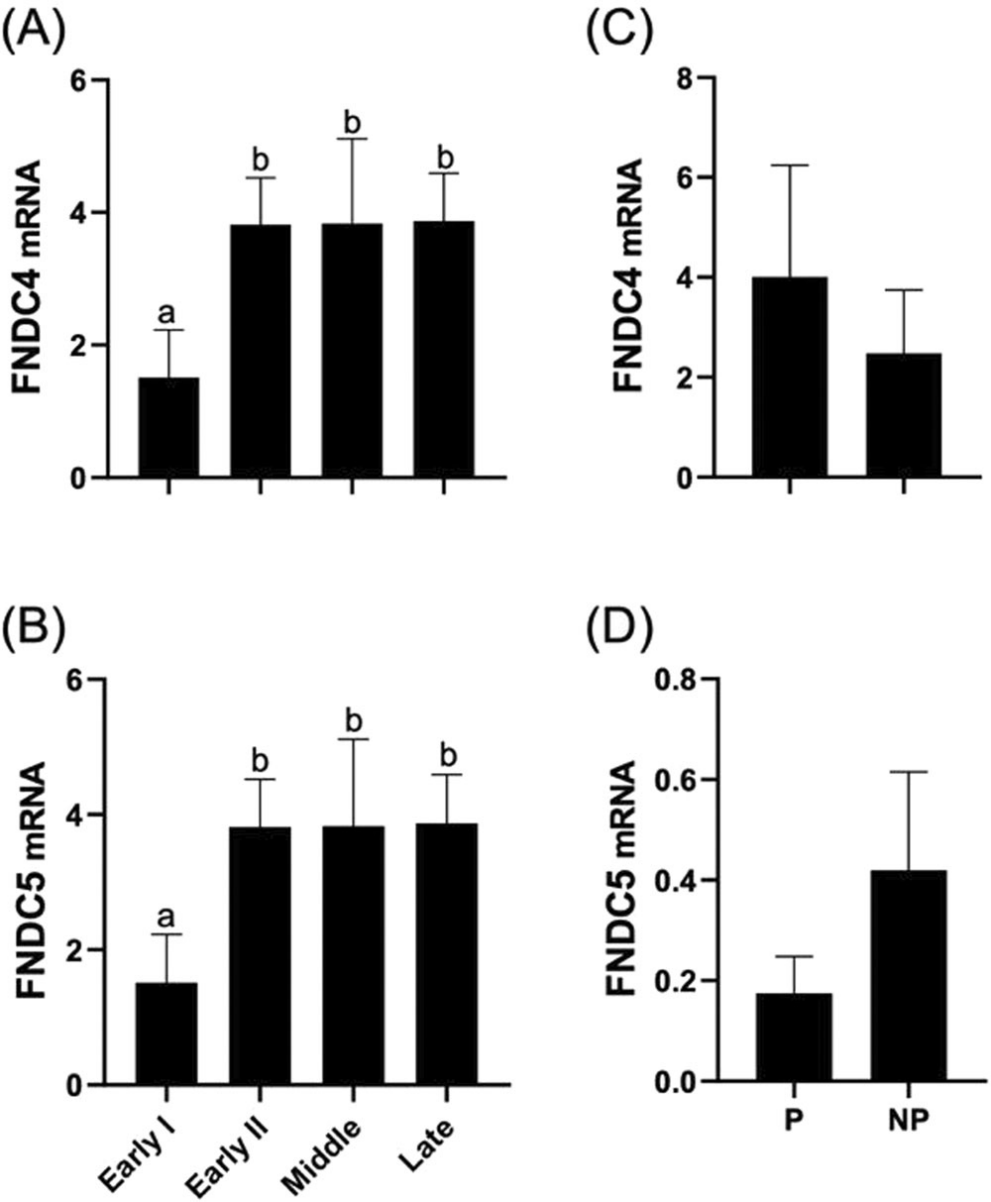
Relative mRNA abundance of *FNDC4* (A), *FNDC5* (B) across different stages of the estrous cycle (Early I, Early II, Middle, Late), and *FNDC4* (C) and *FNDC5* (D) in the corpus luteum of pregnant (P) and nonpregnant (NP) cows on Day 18 post‐insemination. Gene expression was normalized to *GAPDH* and *RPL19*. Different letters indicate significant differences between groups (*p* < 0.05).

The relative abundance of *FNDC4* and *FNDC5* mRNA was significantly greater in Early II, Middle, and Late phase CLs when compared to the Early I CL (Figure 1A,B). When comparing nonpregnant and pregnant CL from cows on Day 18, no difference between groups was found (Figure 1C,D).

### 3.2 ITGAV, ITGB1, and ADGFR5 Expression of mRNA in Bovine CL

**Figure 2:**
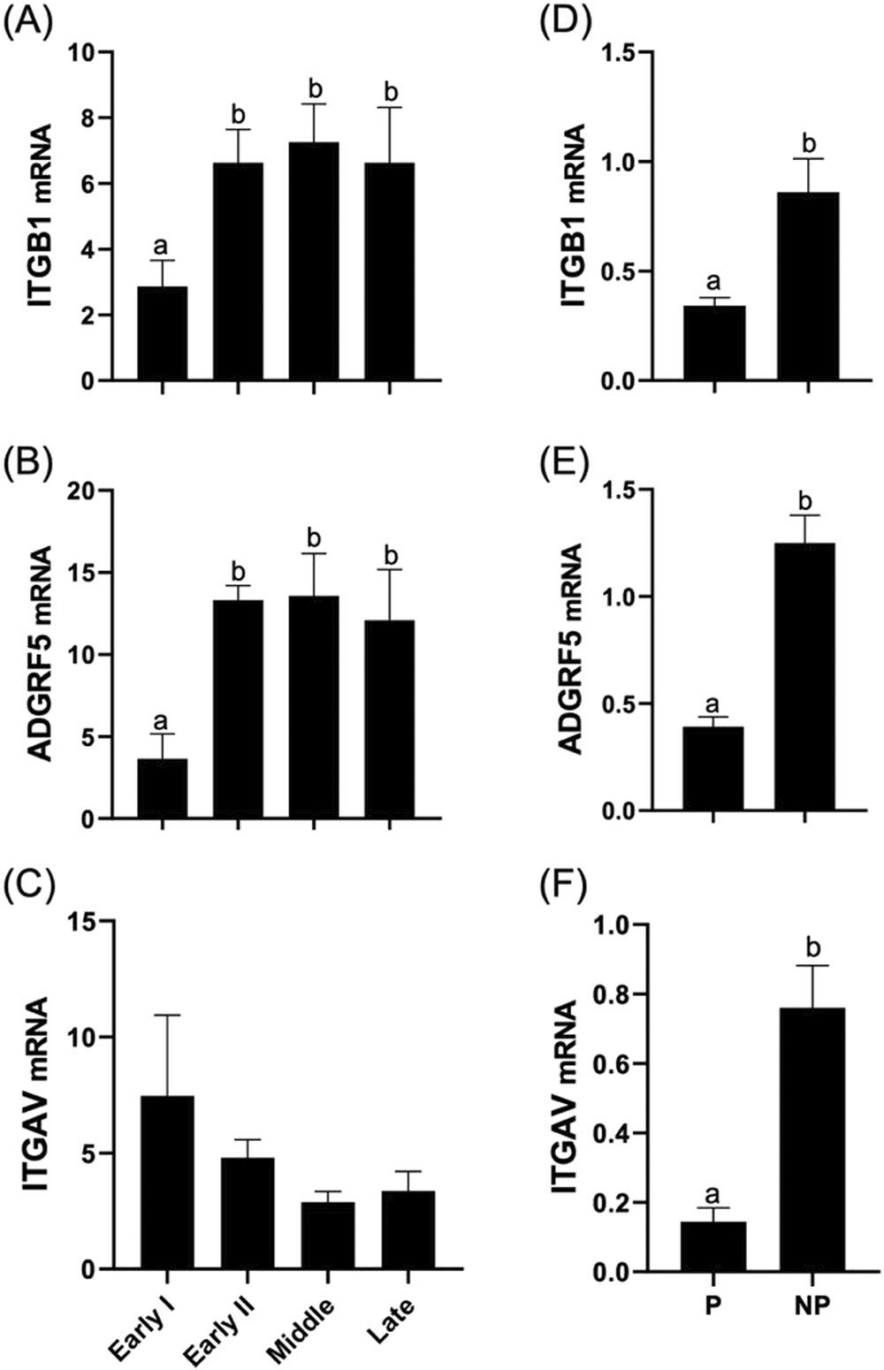
Relative mRNA abundance of *ITGB1* (A), *ADGRF5* (B), and *ITGAV* (C) across different stages of the estrous cycle (Early I, Early II, Middle, Late), and *ITGB1* (D), *ADGRF5* (E), and *ITGAV* (F) in the corpus luteum of pregnant (P) and nonpregnant (NP) cows on Day 18 post‐insemination. Gene expression was normalized to *GAPDH* and *RPL19*. Different letters indicate statistically significant differences between groups (*p* < 0.05).

The expression of *ITGB1* and *ADGRF5* in bovine CL was significantly greater in the Early II, Middle, and Late phases than in the Early I phase (Figure 2A,B), and the abundance of *ITGAV* mRNA did not differ between the phases (Figure 2C). The abundance of mRNA encoding all three receptors was greater in CL of nonpregnant cows on Day 18 compared to pregnant cows (Figure 2D–F).

### 3.3 Steroidogenic Enzyme and Irisin Receptor mRNA Expression in Culture of Bovine Luteal Cells in the Presence or Absence of Irisin and roIFNT

**Figure 3:**
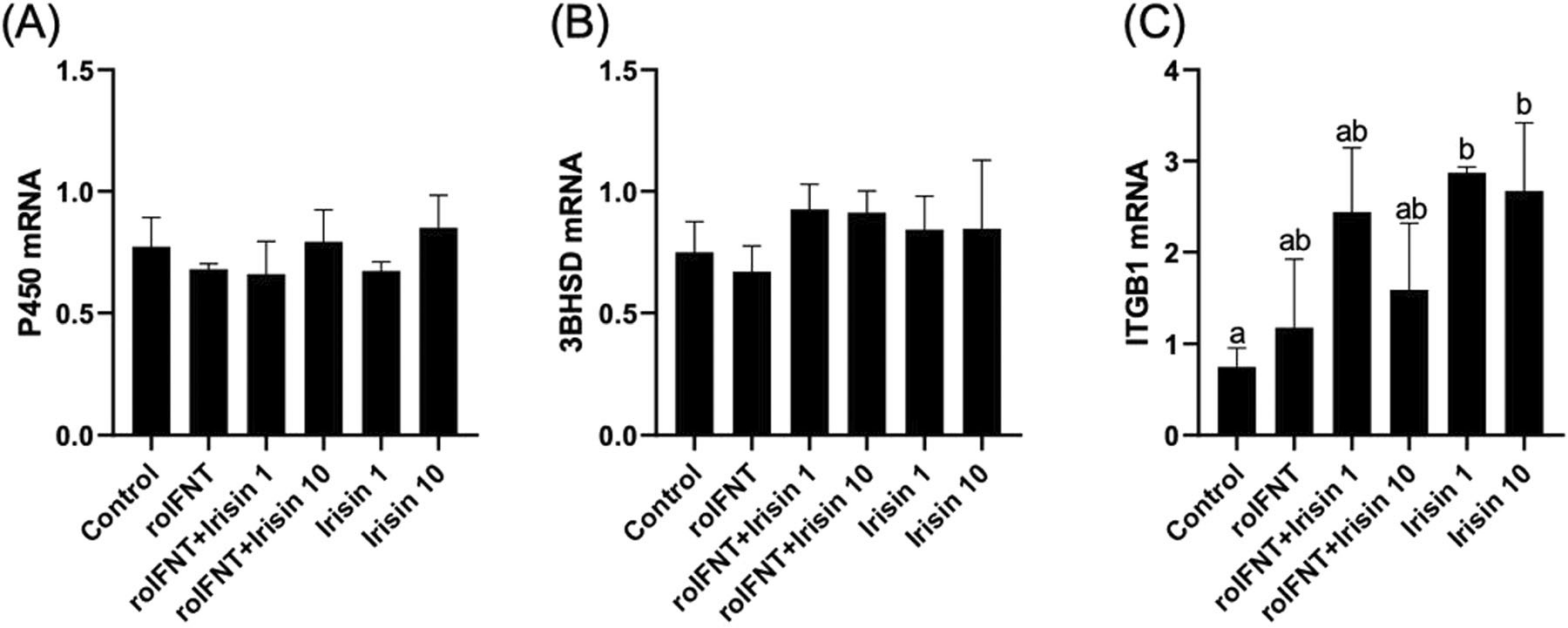
Relative mRNA abundance of steroidogenic genes *P450sCC* (A) and *3βHSD* (B), and the irisin receptor *ITGB1* (C) in luteal cells treated in vitro. Treatment groups: Control, roIFNT (1 ng/mL), roIFNT + irisin (1 or 10 ng/mL), and irisin alone (1 or 10 ng/mL). Different letters indicate significant differences (*p* < 0.05).

Treatment of luteal cells with irisin, irrespective of the presence of roIFNT, had no effect on the abundance of *P450sCC* or *3βHSD* mRNA (Figure 3A,B). Expression of the irisin receptor (*ITGB1*) was increased in the presence of irisin compared to control groups without irisin (Figure 3C).

### 3.4 Irisin Affects IFNT Signaling in Bovine Luteal Cells

**Figure 4:**
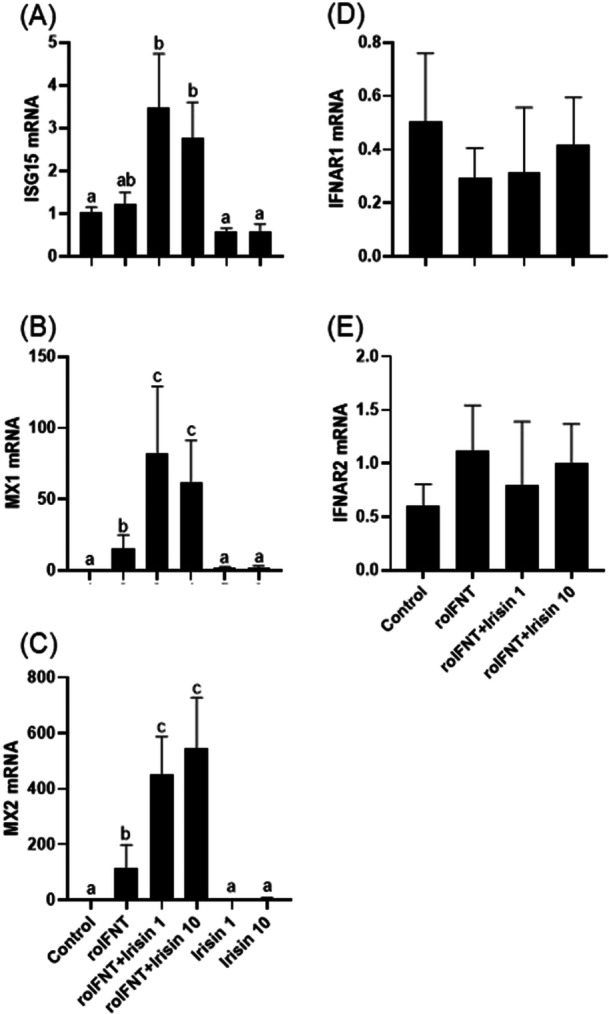
Relative mRNA abundance of interferon‐stimulated genes *ISG15* (A), *MX1* (B), *MX2* (C), and interferon receptors *IFNAR1* (D), *IFNAR2* (E) in luteal cells treated in vitro. Cells were treated with roIFNT (1 ng/mL) and/or irisin (1 or 10 ng/mL). Different letters indicate significant differences (*p* < 0.05).

Neither irisin nor roIFNT has significant effect on *ISG15* mRNA abundance (Figure 4A). The exposure of cells to roIFNT significantly induced the expression of ISGs (*MX1* and *MX2*) (Figure 4B,C), and the treatment with irisin alone had no effect. Pretreatment with irisin significantly increased *MX1* and *MX2* mRNA abundance in response to IFNT. The expression of mRNA of interferon receptors *IFNAR1* and *IFNAR2* was not altered by IFNT or irisin (Figure 4D,E).

### 3.5 Effect of Irisin on Markers of Viability and Proliferation

**Figure 5:**
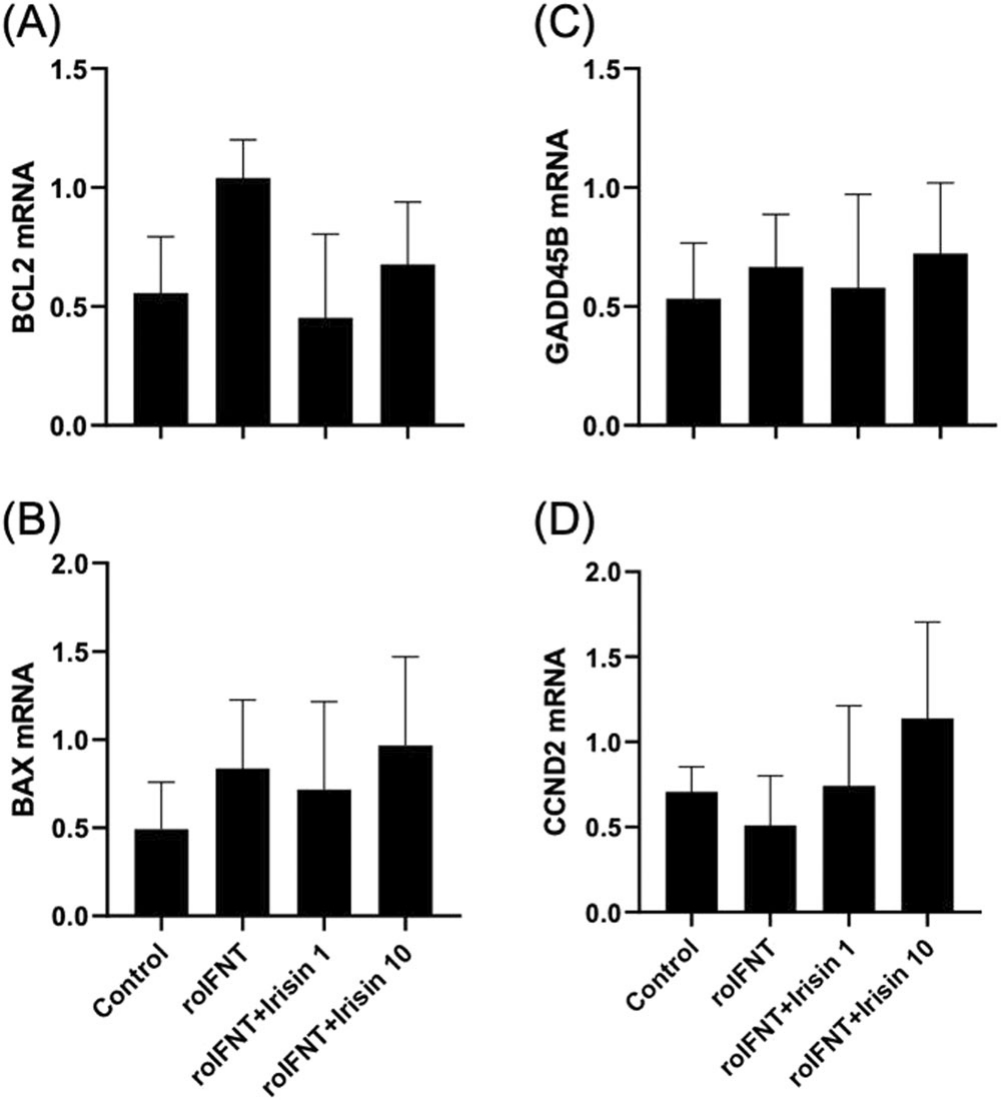
Relative mRNA abundance of genes related to cell viability and proliferation: *BCL2* (A), *BAX* (B), *GADD45B* (C), and *CCND2* (D) in luteal cells treated in vitro. Cells were treated with roIFNT (1 ng/mL) and/or irisin (1 or 10 ng/mL). Different letters indicate significant differences (*p* < 0.05).

Addition of neither irisin or IFNT altered abundance of mRNA of cell cycle (*CCND2*), apoptosis (*BAX* and *BCL2*), or cell stress genes (*GADD45B*) (Figure 5).

## 4 Discussion

The results presented herein describe the presence of FNDC4 and FNDC5 and their receptors *ITGVB1*, *ITGAV*, and *ADGRF5* in the bovine CL. Specifically, we show that the adipokines FNDC4 and FNDC5, both members of the fibronectin type III domain‐containing (FNDC) family, are expressed in the bovine CL, and are abundant in the Middle and Late‐cycle CL compared to the Early I cycle CL. Our results indicate that the adipokine/myokine FNDC5/irisin plays a role in the function of the bovine CL. When investigating the action of irisin on bovine luteal cells, irisin pre‐exposure amplified the stimulatory effects of IFNT on mRNA level of ISGs in vitro.

In the present study, *FNDC4* and *FNDC5* were expressed in the bovine CL, with significantly higher levels in the Early II, Middle, and Late phases of the estrous cycle compared to the Early I phase (Figure 1A,B). These data suggest a dynamic mRNA expression and may be associated with CL functional changes during the estrous cycle. The comparison between CL of pregnant and nonpregnant cows on Day 18 did not reveal significant differences (Figure 1C,D), suggesting that the levels of FNDC4 and FNDC5 may be independent of the reproductive condition at this phase. The receptors for irisin and FNDC4 were also analyzed, and it was observed that *ITGB1* and *ADGRF5* had greater abundance of mRNA in the Early II, Middle, and Late phases than in Early I phase (Figure 2A,B), while *ITGAV* did not change with CL development (Figure 2C). When comparing CL from pregnant and nonpregnant cows on Day 18, all the receptors analyzed had a greater mRNA expression in nonpregnant cows (Figure 2D–F). This pattern may indicate that, during early pregnancy, the receptors are recruited to support CL maintenance, while in nonpregnant cows, they remain more abundant.

Some adipokines are elevated in circulation during the negative energy balance (NEB) period and have been linked to the inhibition of follicular development (Daudon, Ramé, et al. 2022; Daudon et al. 2025). Adding irisin to the culture, we mimic the conditions that occur in vivo in animals experiencing NEB. In production systems, cows must conceive as early as possible after calving to avoid extended calving intervals. However, NEB leads to various adverse changes that reduce fertility and reproductive success (Leroy et al. 2008). During this period, irisin levels are elevated (Daudon, Ramé, et al. 2022), making it essential to understand whether this could influence CL signaling and, consequently, impact reproductive efficiency. According to our findings, irisin modulates IFNT signaling in bovine luteal cells, which may influence the functionality and survival of the CL and consequently the fertility of these animals.

A central finding of our study was the interaction between irisin and IFNT. Exposure to irisin for 12 h before treatment with roIFNT resulted in amplified expression of ISGs (*MX1* and *MX2*), suggesting a role for irisin as a potentiator of IFNT‐mediated signaling in luteal cells. The exposure of luteal cells to irisin before roIFNT treatment is physiologically relevant, as circulating irisin increases in the NEB during the postpartum period and may act on luteal cells before the conceptus‐derived IFNT signaling. This step mimics the temporal dynamics of in vivo exposure and ensure that the observed effects could be attributed to the interaction between irisin and IFNT signaling. On the other hand, irisin alone was unable to induce the expression of these genes, reinforcing that its action is dependent on the presence of roIFNT. Our results showed that the expression of adipokine receptors was significantly greater in CL of nonpregnant cows. One of the main differences between the CL of pregnant and nonpregnant cows at this stage is the exposure to IFNT (Basavaraja et al. 2021; Hughes et al. 2020; Meidan and Basavaraja 2022). The difference observed in pregnant cows, compared to nonpregnant cows, may be associated with receptor occupancy during this period, supporting the hypothesis of an interaction between irisin and IFNT in the CL.

From these results, we investigated whether this response could be related to a greater presence of interferon receptors. Our results show that the expression of *IFNAR1* and *IFNAR2* remained constant between groups (Figure 4D,E), suggesting that the cell's response capability is not limited by increased receptor availability. Also, treatment with irisin appears to increase the expression of its receptor ITGB1 (Figure 3C), but it is present in all groups, suggesting that responsiveness to irisin acts in the same pattern, not limited by receptor availability.

Although irisin altered *MX1* and *MX2* mRNA abundance, neither irisin nor IFNT has an effect on *ISG15*. This may be because the duration of the roIFNT treatment used here may not allow adequate induction of *ISG15*, as prolonged exposure is often necessary for significant mRNA expression (Álvarez et al. 2024).

The interaction between irisin and IFNT appears to be specifically linked to pathways involved in maintaining the CL, without significantly influencing cell proliferation or apoptosis. This conclusion is supported by the observation that expression of genes associated with cell proliferation (*CCND2*) and apoptosis (*BAX*, *BCL2*, *GADD45B*) were not altered in response to irisin and roIFNT. No significant difference was observed in the expression of CCND2, indicating that irisin and roIFNT do not directly modulate cell proliferation pathways (Bott et al. 2010; Bridi et al. 2018). Research in vascular smooth muscle cells has shown that irisin can inhibit proliferation through the HO‐1 pathway, but this effect is context‐dependent and does not extend to all cell types (Liu et al. 2022). The balance between proapoptotic genes (*BAX*) and antiapoptotic genes (*BCL2*) remained unchanged, suggesting that irisin and IFNT do not directly alter apoptosis pathways, and although cellular stress may influence apoptosis, the specific interaction of irisin and IFNT does not seem to significantly involve these pathways (Lamichhane and Samir 2023).

CL maintenance is essential for the continuation of pregnancy and is influenced by complex molecular signals (Wiltbank et al. 2023). The ability of irisin to amplify IFNT signaling suggests a regulatory role in supporting luteal function. This interaction may be particularly relevant in the postpartum period when irisin levels are elevated due to NEB (Daudon, Ramé, et al. 2022). Future studies should explore whether irisin can be used as a strategy to improve fertility in cattle, especially in high‐producing dairy cows. Furthermore, investigating other genes and signaling pathways modulated by irisin and IFNT could expand the understanding of the molecular mechanisms involved.

Although our results provide initial evidence of a potential role for irisin in modulating IFNT signaling, the lack of changes in gene expression does not rule out the possibility of regulation occurring at posttranscriptional or posttranslational levels. It is important to note that mRNA expression does not always correlate with protein abundance or functional activity, which can be influenced by various regulatory mechanisms. Therefore, future studies should include protein‐level analyses to better elucidate the biological effects of irisin on IFNT signaling pathways.

In conclusion, this study demonstrates that the genes FNDC4 and FNDC5 are expressed in the bovine CL at different stages of development and that irisin increases the expression of ISGs in bovine luteal cells in vitro. These findings suggest a potential role for irisin in modulating luteal cell responses to interferon signals. While the mechanisms underlying these effects remain to be fully elucidated, future studies may explore whether irisin contributes to luteal function regulation through indirect pathways, particularly under metabolic conditions such as NEB in the postpartum period.

## Author Contributions

**Ana Paula da Silva:** conceptualization, investigation, writing – original draft, methodology, validation, project administration, visualization, writing – review and editing, formal analysis. **Karine de Vargas Aires:** writing – original draft, investigation, methodology. **Suzana Rossato Feltrin:** writing – original draft, investigation, methodology, validation. **Leonardo Guedes de Andrade:** writing – original draft, investigation, methodology. **Julia Vieira Cambuí:** writing – original draft, investigation, methodology. **Carlos Miguel Staudt:** writing – original draft, investigation, methodology. **Luis Fernando Schütz:** writing – review and editing, writing – original draft. **Christopher Price:** writing – review and editing, writing – original draft. **Valério Marques Portela:** supervision, data curation, funding acquisition, writing – review and editing, formal analysis, project administration, resources, writing – original draft. **Alfredo Quites Antoniazzi:** supervision, resources, writing – review and editing, funding acquisition, project administration, writing – original draft, formal analysis, data curation.

## Conflicts of Interest

The authors declare no conflicts of interest.

## Data Availability

The data that support the findings of this study are available from the corresponding author upon reasonable request.
